# Prevalence of Stroke and Associated Risk Factors in Patients With Atrial Fibrillation: A Cross-Sectional Study

**DOI:** 10.7759/cureus.82915

**Published:** 2025-04-24

**Authors:** Nida Gul, Salma Habib, Felicita M Tayong, Ayaz Ali, Jestin K. J., Nadia Khan, Palwasha Asghar, Khayam, Shah Faisal, Shabnam Shahjehan

**Affiliations:** 1 Medicine, MTI Khyber Teaching Hospital Peshawar, Peshawar, PAK; 2 Internal Medicine, New York Institute of Technology College Of Osteopathic Medicine (NYITCOM), Old Westbury, USA; 3 General Surgery, Tulane University School Of Medicine, New Orleans, USA; 4 College of Medicine, University of Science, Arts and Technology College of Medicine, Brirish West Indies, MSR; 5 Internal Medicine, Khyber Medical College Peshawar, Peshawar, PAK; 6 Pathology and Laboratory Medicine, All India Institute of Medical Sciences, Bilaspur, Bilaspur, IND; 7 Internal Medicine, MTI Khyber Teaching Hospital Peshawar, Peshawar, PAK; 8 Internal Medicine, Combined Military Hospital, Peshawar, PAK

**Keywords:** atrial fibrillation, cha₂ds₂-vasc score, chronic kidney disease, obesity, smoking, stroke

## Abstract

Introduction

Atrial fibrillation (AF) is a common type of heart rhythm disorder that considerably elevates the risk of stroke. Identifying risk factors and their association with stroke in AF patients is essential for effective prevention strategies.

Methodology

This cross-sectional study was conducted at the Cardiology Department of Khyber Teaching Hospital, Peshawar, Pakistan, from October 2024 to March 2025. A total of 345 patients with diagnosed AF were enrolled using a non-probability purposive sampling technique. The CHA₂DS₂-VASc (Congestive heart failure, Hypertension, Age ≥75 years, Diabetes Mellitus, Prior Stroke or transient ischemic attack (TIA) or thromboembolism, Vasculardisease, Age 65-74 years, Sex category) score was used to assess stroke risk, and associations with various risk factors were analyzed using the Chi-square test.

Results

Stroke or transient ischemic attack (TIA) was reported in 13% of patients. Significant associations were found between stroke risk and smoking, obesity, chronic kidney disease, thyroid disease, and physical inactivity. No significant correlation was found with alcohol consumption, likely due to cultural and religious practices.

Conclusion

This study highlights a notable stroke prevalence among AF patients and underscores the importance of managing modifiable risk factors to reduce stroke risk.

## Introduction

It is estimated that approximately 33 million individuals globally are affected by atrial fibrillation (AF) [1,2]. The actual number of cases may be higher, as many individuals remain unaware of their atrial fibrillation until symptoms appear or they experience complications such as ischemic stroke or systemic thromboembolism. The growing prevalence of AF is largely attributed to an aging population and the rising incidence of risk factors like hypertension, diabetes, obesity, and alcohol use [3].

Stroke represents one of the most serious complications associated with atrial fibrillation (AF). Approximately one-quarter of all ischemic strokes are of cardioembolic origin, with AF being the leading underlying cause [4]. The risk of stroke specifically linked to atrial fibrillation rises with advancing age, in contrast to other risk factors like hypertension, which do not show the same age-related increase [5].

Individuals with atrial fibrillation (AF) often experience bothersome symptoms due to hemodynamic irregularities; however, the most significant concern is the elevated risk of cerebrovascular events such as transient ischemic attacks (TIA) and ischemic strokes. AF contributes to this risk by promoting thrombus formation within the left atrium. If these clots become dislodged, they can travel to the brain and block cerebral blood flow, resulting in a thromboembolic stroke [6].

Identifying atrial fibrillation is crucial for preventing stroke, as the use of anticoagulant therapy has been proven to reduce the risk of stroke in patients with AF [7]. Studies have shown that antiplatelet therapy cannot serve as an effective substitute for appropriate anticoagulation in patients with atrial fibrillation [8]. In individuals who do not have atrial fibrillation, stroke prevention, when needed, typically involves the use of antiplatelet medications [9].

The decision to initiate anticoagulation in atrial fibrillation is typically based on clinical risk assessment tools such as the CHA₂DS₂-VASc (Congestive heart failure, Hypertension, Age ≥75 years, Diabetes Mellitus, Prior Stroke or TIA or thromboembolism, Vascular disease, Age 65-74 years, Sex category) score. This scoring system takes into account factors like heart failure, hypertension, age (categorized into two groups), diabetes, previous stroke, vascular disease, and sex. The presence of one or more of these risk factors generally supports the use of oral anticoagulant therapy [10].

A study was conducted in Pakistan, which showed a stroke prevalence of 1.2% [11].

The objective of this study is to assess the prevalence of stroke in patients with atrial fibrillation and to evaluate the association between stroke risk and various contributing risk factors within this population.

## Materials and methods

This cross-sectional study was conducted over a six-month period, from October 2024 to March 2025, in the Cardiology Department of Khyber Teaching Hospital, Peshawar. Approval for the study was secured from two primary institutional authorities: the Ethical Review Committee of Khyber Medical College, Peshawar, and the Office of the Medical Director at Khyber Teaching Hospital.

A non-probability purposive sampling technique was used for data collection. Patients diagnosed with atrial fibrillation who visited the cardiology ward or outpatient department of Khyber Teaching Hospital during the study period and provided informed consent were included. Individuals who did not meet these criteria were excluded. In total, 345 participants fulfilling the inclusion criteria were enrolled in the study.

Stroke was defined based on a prior confirmed diagnosis, verified through hospital records and imaging reports. In cases where documentation was unavailable, clinical history was considered. Obesity was assessed by calculating body mass index (BMI), and those with a BMI greater than 30 were classified as obese. The presence of chronic kidney disease (CKD) and thyroid disorders was confirmed through review of the patients’ existing medical records. 

Data analysis

SPSS (Statistical Package for the Social Sciences) 22 software (IBM Corp., Armonk, USA) was utilized to perform statistical analysis on the collected data. The CHA₂DS₂-VASc score was used to assess the stroke risk of patients with atrial fibrillation. The Chi-square test was applied to determine the statistical significance of associations between various stroke risk factors and stroke risk levels among the participants. The chi-square test was considered significant when the p-value was less than 0.05. Data was presented in the form of frequency tables.

## Results

A total of 345 patients diagnosed with atrial fibrillation were included in the study. These patients were either admitted to the cardiology ward or attended the cardiology outpatient department at Khyber Teaching Hospital during the study period and provided informed consent to participate. Most of the participants were male, comprising 264 individuals (76.52%) of the sample, whereas females made up 81 participants (23.48%). Most of the patients were under 65 years of age, with a smaller proportion aged between 65 and 74 years, and a few aged above 75 years. The detailed age distribution of the study population is presented in Table 1.

**Table 1:** Age-wise distribution

Age	Frequency (n)	Percentage (%)
<65 years	288	83.5
65-74 years	51	14.8
>75 years	6	1.7
Total	345	100

**When patients were asked about their educational background, the majority, 201 (58.25%)**, reported having no formal education. The detailed distribution of education levels among the participants is provided in Table 2.

**Table 2:** Education of the participants

	Categories	Participants’ Gender		Total
		Male n (%)	Female n (%)	
Education of Participants	Primary	38 (11.01)	7 (2.03)	45
	Secondary	40 (11.59)	28 (8.12)	68
	Tertiary	20 (5.79)	11 (3.19)	31
	Uneducated	166 (48.11)	35 (10.14)	201

Assessment of the CHA₂DS₂-VASc score revealed that the majority of patients fell in the moderate risk category. During the early validation studies, individuals scoring 0 on the CHA₂DS₂-VASc scale were identified as low risk, those with scores of 1 to 2 were labeled as moderate risk, and scores above 2 indicated a high risk category (Table 3) [12].

**Table 3:** CHA2DS2-VASc scoring CHA2DS2-VASc: Congestive heart failure, Hypertension, Age ≥75 years, Diabetes Mellitus, Prior Stroke or transient ischemic attack (TIA) or thromboembolism, Vascular disease, Age 65–74 years, Sex category

Risk (CHA2DS2 VASc Score)	Frequency (n)	Percentage (%)
Low Risk (0 score)	111	32.2
Moderate Risk (1-2 score)	218	63.2
High Risk (≥3)	16	4.6

Out of the 345 patients enrolled in the study, 45 (13%) had previously experienced a stroke or transient ischemic attack (TIA), reflecting a stroke prevalence of about 13% among individuals diagnosed with atrial fibrillation.

The Chi-square test was applied to evaluate the association between various stroke risk factors in atrial fibrillation patients and the CHA₂DS₂-VASc score. A statistically significant association was observed with several factors, including smoking (p < 0.001), obesity (p < 0.001), decreased physical activity (p = 0.02), chronic kidney disease (CKD) (p = 0.02), and thyroid disease (p = 0.03). However, no significant association was found with alcohol consumption (p = 0.428).

A significant relationship was also identified between the duration of atrial fibrillation and stroke risk. Most patients diagnosed within the past year were found to be at moderate risk (p < 0.001). Additionally, patients on medication for atrial fibrillation exhibited a higher stroke risk compared to those who had undergone pacemaker insertion or radiofrequency ablation (Table 4).

**Table 4:** Risk factors of stroke CHA2DS2-VASc: Congestive heart failure, Hypertension, Age ≥75 years, Diabetes Mellitus, Prior Stroke or transient ischemic attack (TIA) or thromboembolism, Vascular disease, Age 65–74 years, Sex category

Risk Factors		CHA2DS2-VASc Score			P-value (significant when <0.05)	Chi-square Value
		Low risk	Moderate Risk	Severe Risk		
Smoking	Yes	109	73	0	<0.001	148.031
	No	2	145	16		
Alcohol Consumption	Yes	0	3	0	0.428	3.843
	No	111	215	16		
Obesity	Yes Obese	54	173	16	<0.001	41.540
	Not Obese	57	45	0		
Chronic Kidney Disease	Yes	24	73	2	0.024	11.145
	No	87	145	14		
Thyroid Diseases	Yes	24	73	2	0.034	11.245
	No	87	145	14		
Atrial Fibrillation Diagnosis	<1 year	45	86	2	<0.001	38.770
	1-5 years	23	67	11		
	6-10 years	20	32	1		
	>10 years	23	33	2		
Atrial Fibrillation Treatment	Medication	36	100	5	<0.001	33.336
	Radiofrequency Ablation	10	43	7		
	Pacemaker Insertion	65	75	4		
Physical Activity	Yes	109	73	0	0.002	148.031
	No	2	145	16		

## Discussion

In our study, we gathered data from a cohort of 345 patients diagnosed with atrial fibrillation. The majority of participants were male, and most were under the age of 65 years, which is noteworthy, as AF is traditionally more prevalent in older age groups. This demographic distribution may reflect either early-onset AF or improved diagnostic efforts in younger populations.

The study revealed that approximately 13% of individuals with atrial fibrillation had a prior history of stroke. This finding is consistent with previous research, such as a study conducted in Indonesia reporting a 10.8% prevalence of stroke in AF patients [13]. However, stroke prevalence in AF populations has shown considerable variability across the literature, with some studies reporting rates as high as 19.4% [14] while some showing a prevalence of 3.4% [15]. These discrepancies may stem from differences in study design, sample size, and population characteristics, as well as varying degrees of access to preventive healthcare, including anticoagulation therapy.

Our study identified a significant association between smoking and the CHA₂DS₂-VASc score, which is a well-established tool used to assess stroke risk in patients with AF. This correlation supports findings in earlier literature and highlights smoking as a modifiable risk factor that directly contributes to increased cardiovascular and thromboembolic risks [16]. Interestingly, in contrast to studies that reported a significant link between alcohol consumption and elevated CHA₂DS₂-VASc scores [17], our analysis did not find such an association. This deviation is likely due to the cultural and religious composition of our study population, where alcohol consumption is often limited or entirely absent due to religious prohibitions. Such sociocultural factors must be carefully considered when evaluating lifestyle-related risk contributors in clinical research.

We also found a significant association between CKD and increased stroke risk among AF patients. This finding is consistent with a growing body of evidence that positions CKD as an independent risk factor for adverse cardiovascular outcomes in AF [18,19]. CKD contributes to systemic inflammation, endothelial dysfunction, and abnormalities in coagulation, all of which may amplify the propensity for thromboembolic complications. Therefore, early detection and rigorous management of CKD in patients with AF could substantially reduce stroke incidence in this vulnerable group.

In addition, our results indicated a notable association between obesity and stroke risk in AF patients. Obesity is increasingly recognized as a driver of systemic inflammation and a prothrombotic state, which can exacerbate atrial remodeling and increase the risk of thromboembolic events [20]. The presence of obesity may also coexist with other metabolic risk factors such as hypertension, diabetes, and dyslipidemia, further compounding the risk. These observations emphasize the necessity of a comprehensive risk assessment approach that includes metabolic and lifestyle factors in AF management.

Limitations

There are certain limitations in this study that need to be acknowledged. Firstly, it was conducted at a single center, which may limit the generalizability of the findings to other populations or clinical settings. Secondly, the use of a non-probability sampling technique may introduce selection bias, affecting the representativeness of the sample. Finally, due to the cross-sectional nature of the study, it is not possible to determine a causal relationship between the observed risk factors and the prevalence of stroke in individuals with atrial fibrillation.

## Conclusions

This study highlights a 13% prevalence of stroke or TIA among patients with atrial fibrillation, with most patients falling into the moderate risk category based on the CHA₂DS₂-VASc score. Significant associations were observed between stroke risk and factors such as smoking, obesity, CKD, thyroid disease, and physical inactivity. Alcohol consumption showed no significant correlation, likely due to cultural factors. These findings underscore the importance of comprehensive risk assessment and targeted interventions to prevent stroke in AF patients.
